# Structure-based epitope prediction and assessment of cross-reactivity of *Myrmecia pilosula* venom-specific IgE and recombinant Sol g proteins (*Solenopsis geminata*)

**DOI:** 10.1038/s41598-024-61843-4

**Published:** 2024-05-15

**Authors:** Hathairat Buraphaka, Theerawat Dobutr, Michael D. Wiese, Andreas L. Lopata, Sakda Daduang

**Affiliations:** 1 Faculty of Pharmaceutical Sciences, Khon Kaen University, Khon Kaen, Thailand; 2 Protein and Proteomics Research Center for Commercial and Industrial Purposes (ProCCI), Khon Kaen University, Khon Kaen, Thailand; 3 Department of Biomedical Sciences and Biomedical Engineering, Faculty of Medicine, Prince of Songkla University, Hat Yai, Thailand; 4 Centre for Pharmaceutical Innovation, UniSA: Clinical and Health Sciences, University of South Australia, Adelaide, Australia; 5 Molecular Allergy Research Laboratory, Australian Institute of Tropical Health and Medicine, James Cook University AU, Townsville, Australia; 6 Tropical Futures Institute, James Cook University SG, Singapore, Singapore

**Keywords:** Computational models, Protein function predictions, Protein structure predictions, Biochemistry, Risk factors

## Abstract

The global distribution of tropical fire ants (*Solenopsis geminata*) raises concerns about anaphylaxis and serious medical issues in numerous countries. This investigation focused on the cross-reactivity of allergen-specific IgE antibodies between *S. geminata* and *Myrmecia pilosula* (Jack Jumper ant) venom proteins due to the potential emergence of cross-reactive allergies in the future. Antibody epitope analysis unveiled one predominant conformational epitope on Sol g 1.1 (PI score of 0.989), followed by Sol g 2.2, Sol g 4.1, and Sol g 3.1. Additionally, Pilosulin 1 showed high allergenic potential (PI score of 0.94), with Pilosulin 5a (PI score of 0.797) leading in B-cell epitopes. The sequence analysis indicated that Sol g 2.2 and Sol g 4.1 pose a high risk of cross-reactivity with Pilosulins 4.1a and 5a. Furthermore, the cross-reactivity of recombinant Sol g proteins with *M. pilosula*-specific IgE antibodies from 41 patients revealed high cross-reactivity for r-Sol g 3.1 (58.53%) and r-Sol g 4.1 (43.90%), followed by r-Sol g 2.2 (26.82%), and r-Sol g 1.1 (9.75%). Therefore, this study demonstrates cross-reactivity (85.36%) between *S. geminata* and *M. pilosula*, highlighting the allergenic risk. Understanding these reactions is vital for the prevention of severe allergic reactions, especially in individuals with pre-existing Jumper Jack ant allergy, informing future management strategies.

## Introduction

The ants belonging to the *Solenopsis* genus are stinging insects classified under the order Hymenoptera, commonly referred to as "Fire ants". All species within the *Solenopsis* genus exhibit a highly aggressive nature and possess a venomous sting that induces a burning sensation^1^. Their venom, delivered through stingers capable of multiple stings, can be injected into their victims^2^. Over 200 species of *Solenopsis* have been identified, with four species, namely *S. invicta*, *S. richteri*, *S. geminata*, and *S. saevissima*, recognized for their medical significance^3,4^. *S. geminata*, also known as "Tropical fire ants," are a harmful invasive species predominantly found in tropical regions. The venom of fire ants consists of 90–95% water-insoluble alkaloids (piperidines), primarily 2-methyl-6-alkyl piperidines and 2-methyl-6-alkenyl piperidines, with approximately 0.1% (w/w) being proteinaceous^5–7^. Tropical fire ants (TFAs) contain four allergenic proteins: Sol g 1, Sol g 2, Sol 3, and Sol g 4, most of which are related to allergic proteins of *S. invicta* (Sol i proteins)^8^. Sol g 1 is a phospholipase A1 and is part of the lipoprotein lipase family, bearing similarities to a protein found in wasp venom. Sol g 2 and Sol g 4 share structural homology, with their biological function(s) remaining elusive. Sol g 3, belongs to the antigen 5 protein family and its biological function is yet to be determined. A single sting from a fire ant is sufficient to trigger the production of specific IgE antibodies, even though it contains only 10–100 ng of protein^9^. Furthermore, the distribution of TFAs has been influenced by human activities such as transportation, mating flights, and colonies floating in floodwater. Additionally, *S. geminata* is a significant species of stinging ant that has been documented as a cause of anaphylaxis in various Asian islands, such as Indonesia and Taiwan^10^. The three major allergens in TFA venom capable of triggering an IgE-mediated allergy are 26, 55, and 75 kDa proteins. However, the dispersal of TFAs is mainly confined to tropical and sub-tropical regions worldwide^11–13^. The TFAs have extended their presence to Northern Australia (including the Northern Territory, Queensland, and Northwestern) and Southwestern Australia^14,15^. The distribution of TFAs in Australia nowadays is primarily influenced by the mating flights of newly mated queens^16^.

In Australia, *Myrmecia pilosula*, commonly known as the ‘jack jumper’ or ‘hopper’ ant, is a native species primarily found in Southeast Australia^17^. It is known to frequently induce allergic reactions in individuals, with documented cases of fatalities resulting from anaphylactic reactions^18^. The venom is primarily composed of five families of low molecular weight peptides with high basicity, collectively known as Pilosulins or Myr p. These peptides, Pilosulin 1, Pilosulin 2, Pilosulin 3, Pilosulin 4, Pilosulin 5, possess distinct characteristics and exhibit limited structural similarity to peptides found in the venom of another Hymenoptera^19–22^. Pilosulins (*M. pilosula*) are peptides with biological activity, demonstrating cytotoxic, hypotensive, histamine-releasing, and antimicrobial properties^19^. Importantly, Wiese et al. (2007) reported that [Ile^5^] Pilosulin 1 (a natural variant form of Pilosulin 1), Pilosulin 3, and Pilosulin 4.1 serve as allergens and are identified by IgE in 33.3%, 77.7%, and 16.7% of sera from individuals allergic to *M. pilosula* venom, respectively^23^.

Cross-reaction within *Solenopsis* spp. was reported, indicating that the whole venom extract of *S. geminata* can yield positive results when detecting *S. invicta*-specific IgE from patients' sera^24^. There is no report on the phenomenon of cross-reactivity between *Solenopsis* spp. allergenic proteins from different genera of ants. Allergen cross-reactivity occurs when IgE antibodies can attach to comparable or nearly identical surface regions of another closely related allergenic protein^25,26^. The molecular basis of allergic cross-reactivity lies in the structural similarity shared by proteins from various sources^27^. It is also possible in protein sequences with high identity, defined as exceeding 70%^25^. Many factors influence cross-reactivity, such as the immune response to the allergen in a host, the level of exposure to the allergen, and the type of allergen^27^. While numerous reports exist on IgE cross-reactivity among Hymenoptera venoms^28,29^, there is a lack of information concerning the potential cross-reaction between *S. geminata* and *M. pilosula* ant venom. Therefore, this study aims to predict cross-reactions among unrelated allergens from ant venoms using an in silico approach and assessing the cross-reactivity of recombinant Sol g proteins with *M. pilosula*-specific IgE via the immunoblotting method.

## Results

### Sequence analysis

**Figure 1:**
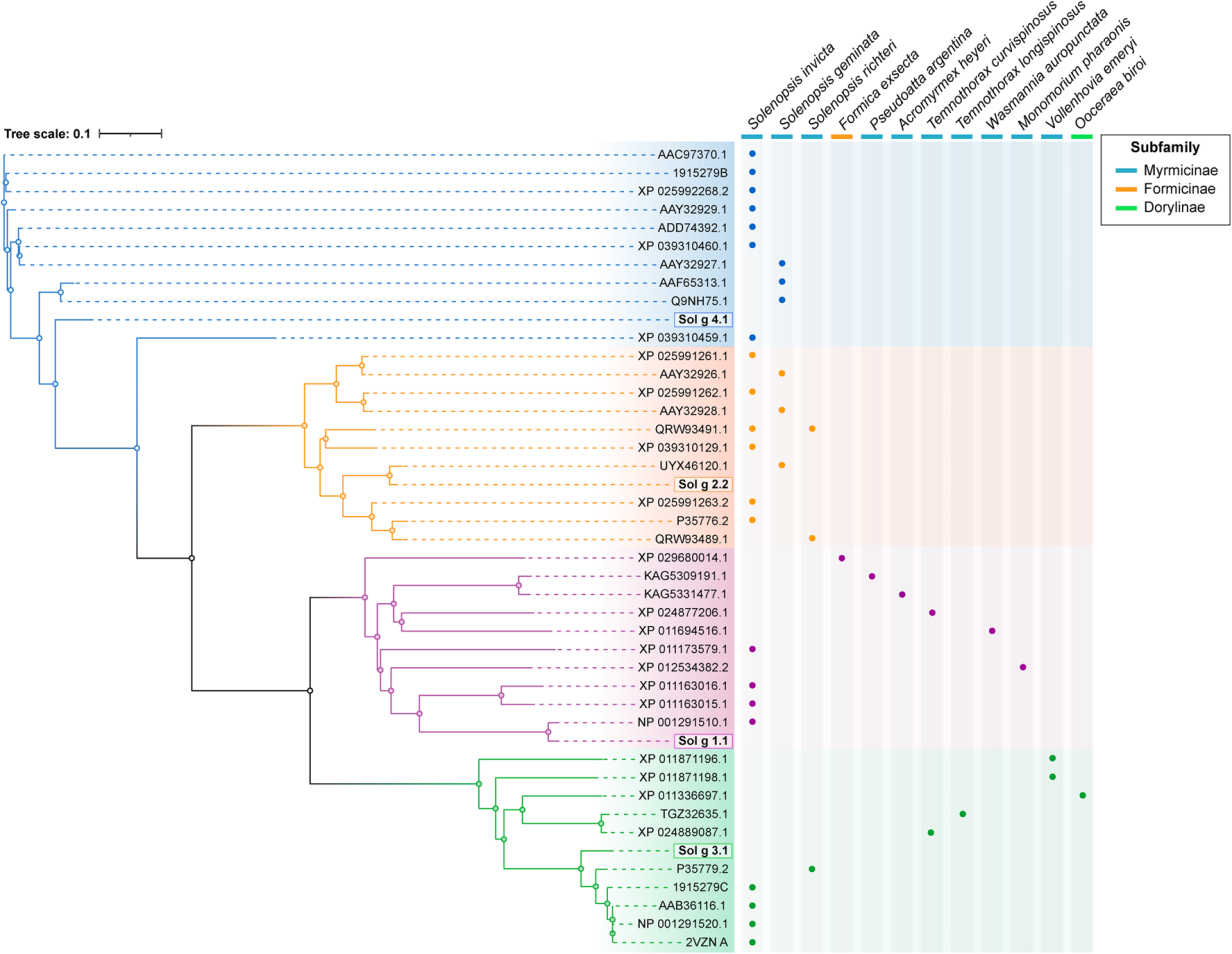
The phylogenetic tree of Sol g proteins, along with their top 10 similarity sequences and associated ant species, was constructed based on the NCBI database. The Clustal Omega tool was employed to generate the phylogenetic tree, and data groups, represented by accession numbers, were highlighted in specific colors: blue for Sol g 4.1, orange for Sol g 2.2, purple for Sol g 1.1, and green for Sol g 1.1. Additionally, three subfamilies were identified with light navy blue indicating Myrmicinae, orange: Formicinae, and light green: Dorylinae.

The deduced amino acid sequence of Sol g proteins was examined using multiple sequence alignment (MSA) to identify the top 10 protein similarities in the NCBI database. The data were organized into four phylogroups, representing homologous protein sequences of Sol g 4.1 (depicted in blue), Sol g 3.1 (depicted in orange), Sol g 1.1 (depicted in purple), and Sol g 2.2 (depicted in green). The phylogenetic tree illustrated that the homologous protein sequences of Sol g 4.1 closely resembled those of Sol g 2.2, along with their respective homologous sequences (see Fig. 1). The venom allergen 4 of *S. invicta* (XP_039310459.1), which exhibited high similarity to the Sol g 4.1 protein sequence, also shared a greater degree of similarity with the Sol g 2.2 protein sequence compared to other Sol g proteins. The Sol g 2.2 protein sequence was associated with the hydrophobic cavity-binding protein of *S. geminata* (UXY46120.1). Additionally, the Sol g 1.1 and 3.1 protein sequences were highly correlated with the phospholipase A1 precursor of *S. invicta* (NP_001291510.1) and venom allergen 3 of *S. richteri* (P35779.2), respectively. Importantly, both Sol g 1.1 and 3.1 protein sequences showed strong cross-relations with sequences from diverse species, whereas the Sol g 2.2 and 4.1 protein sequences were notably specific to *Solenopsis* spp. only.

### Prediction of the three-dimensional (3D) Structure of sol g protein and pilosulin proteins

**Figure 2:**
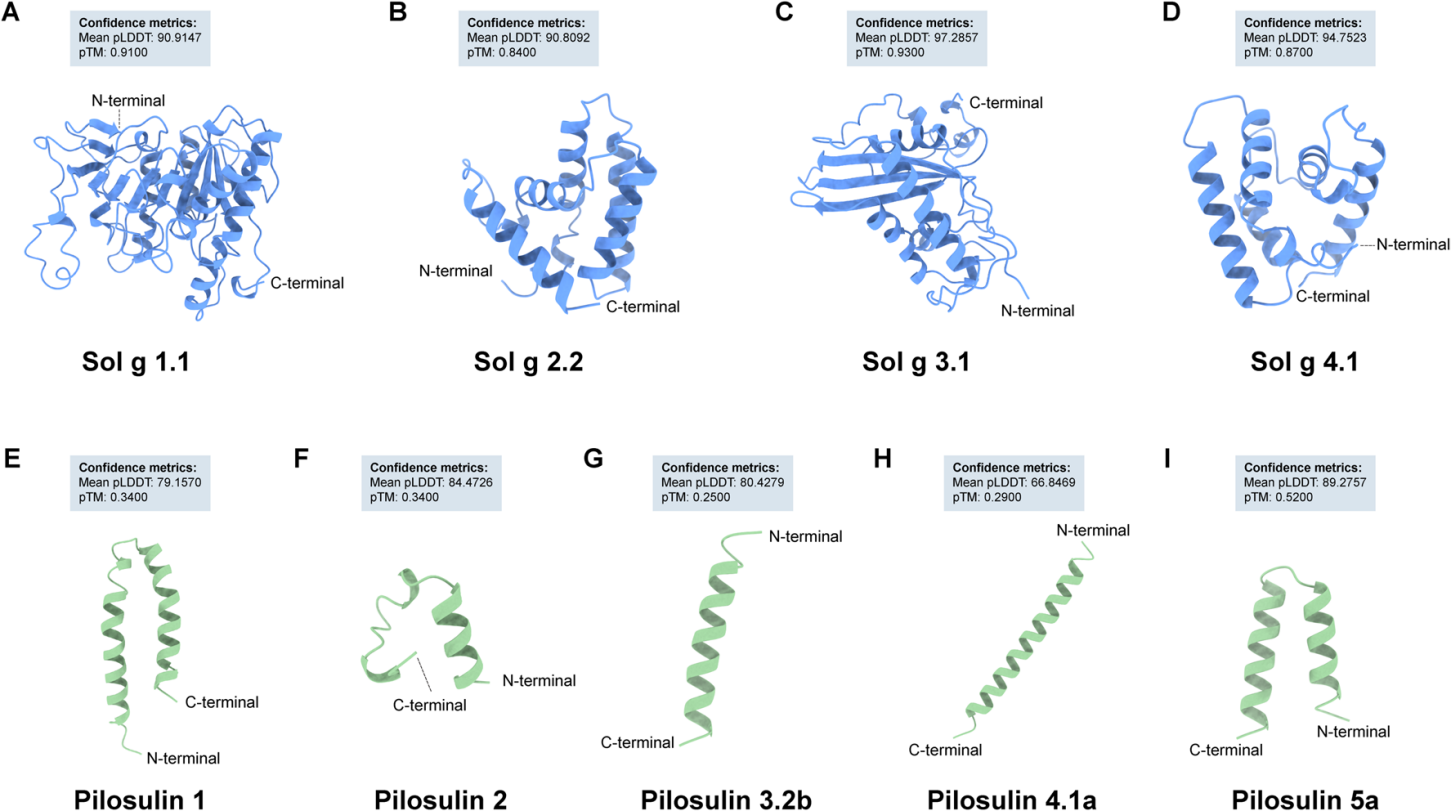
The AlphaFold predictions for the structures of (**A**–**D**) Sol g proteins and (**E**–**I**) Pilosulins are displayed. These top-ranking predictions represented the most confident metrics, such as the pLDDT and pTM scores for each model.

We employed RT-PCR, PCR, and standard cloning techniques to acquire the complete cDNA sequences of *S. geminata* venom allergens, designated as Sol g 1.1, 2.2, 3.1, and 4.1 protein. The mature protein sequences (excluding the signal peptide) of Sol g and Pilosulin proteins were utilized for predicting their 3D structures using AlphaFold, conducted between September 20 and 28, 2023. The majority of Sol g protein 3D structures exhibited a high-confidence pLDDT score exceeding 90, along with pTM > 0.84 (Supplementary File-Figs. 1A,B, 2A,B). Among these, the 3D structure prediction of Sol g 3 displayed the highest confidence score, with a mean pLDDT of 97.2857 and pTM of 0.93 (Fig. 2A–D). Additionally, the pLDDT plot indicated low confidence (pLDDT 66.84–84.47) in the scores of residues for Pilosulin proteins, along with their corresponding pTM scores (Fig. 2E–I).

The features of Sol g models were assessed by comparing their precision structures with experimental structures in SWISS-MODEL. Our analysis revealed that Sol g 1.1 exhibits a high homology (sequence identity = 98.06%) with the structure of Phospholipase A1—*Solenopsis invicta* (PDB ID: Q68KK0). The lipase domain, highlighted in purple on its structure (Asp33-Trp214), displays active sites at Ser130, Asp150, and His221. The Sol g 3.1, homologous to P35778.1.A. Venom allergen 3 (sequence identity = 91.04%), shares the cysteine-rich secretory proteins, antigen 5, and pathogenesis-related 1 protein (CAP) domain within the Ala 47—Tyr 196 region of its protein sequence. Additionally, both Sol g 2.2 and 4.1 models exhibit structural similarities, forming a configuration consisting of five α-helices (Supplementary File- Figs. 1A,B, 2A,B). However, Sol g 2.2 and 4.1 do not belong to identifiable protein families.

### In silico analysis of potential allergenicity

#### Discontinuous B-cell epitope prediction using ElliPro (antibody epitope prediction)

**Figure 3:**
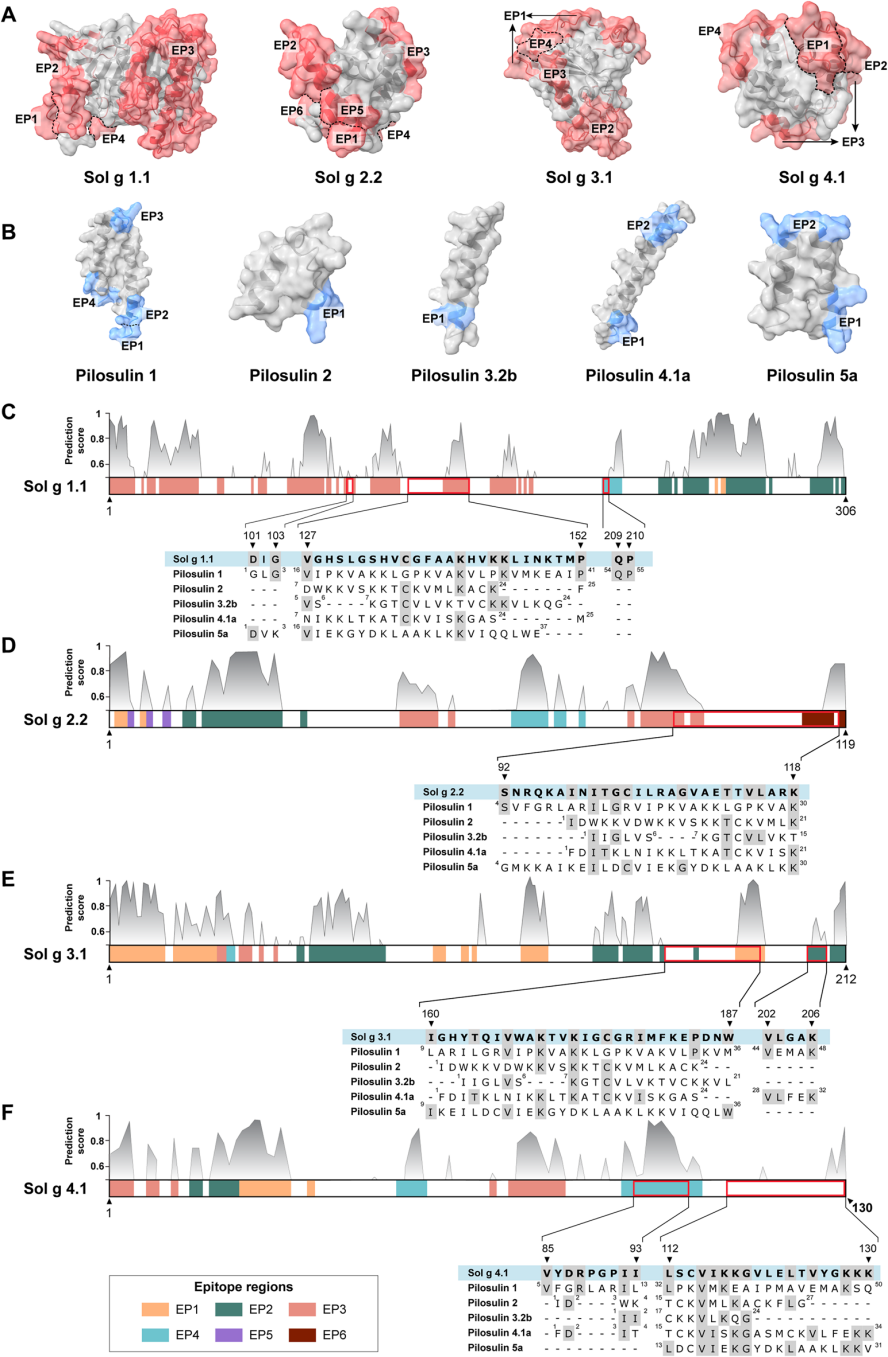
The surface mapping of conformation B-cell epitopes for (**A**) Sol g proteins and (**B**) Pilosulins was obtained from ElliPro, an antibody epitope prediction tool, utilizing 3D structure information in PDB format. (**C**–**F**) Different colors were used to map the schematic diagram of Sol g proteins, indicating the predicted B-cell epitopes. In the schematic diagram, peaks depicted in a grey gradient color represent potential B-cell epitopes with scores exceeding 0.5 (considered as threshold values). The sequence alignment below the schematic diagram highlights identical amino acid residues, marked in a dark grey color, shared between Sol g protein and Pilosulins.

Antigenic protein surface structures were predicted to screen for conformational epitopes in Sol g proteins (Table 1) and Pilosulins (Table 2). ElliPro prediction identified epitope regions composed of amino acid residues associated with positions ranked by PI score. The predominant conformational epitope on the surface protein structure was observed in Epitope 1 of Sol g 1.1, predicted with a PI score of 0.989, followed by Sol g 2.2 (PI score of 0.88), Sol g 4.1 (PI score of 0.773), and Sol g 3.1 (PI score of 0.739). Additionally, the 3D model representation of discontinuous B-cell epitopes was shaded in pale red and pale blue to indicate the allergenicity locations of Sol g proteins and Pilosulins, respectively (Fig. 3A,B). Notably, Sol g 2.2 exhibited a high allergic potential for B-cell epitopes with six positions. The β1 loop of Sol g 2.2 structure showed high allergenicity, consisting of three regions, including Epitope 1 (PI score = 0.83, length = 3 aa), Epitope 3 (PI score = 0.723, length = 17 aa), and Epitope 5 (PI score = 0.596, length = 3 aa).

**Table 1:** The discontinuous B-cell epitope Prediction of Sol g proteins from ElliPro^30^.

Protein names	Epitope no	Residues	Number of residues	PI^a^ Score
Sol g 1.1	Epitope 1	A:W257, A:V259, A:F260	3	0.989
	Epitope 2	A:S233, A:G234, A:R235, A:C236, A:Q237, A:L239, A:W243, A:T244, A:A245, A:Q246, A:Q247, A:R248, A:I249, A:N250, A:P251, A:I252, A:Q253, A:T261, A:S262, A:N263, A:I264, A:P265, A:A266, A:Y267, A:P268, A:T269, A:S270, A:D271, A:T272, A:K273, A:N274, A:C275, A:V276, A:V277, A:N279, A:F296, A:P297, A:D298, A:C299, A:A300, A:Q301, A:N302, A:L303, A:F304, A:C306, A:Q308, A:Q309	47	0.717
	Epitope 3	A:Q1, A:S2, A:C3, A:V4, A:Y5, A:G6, A:N7, A:S8, A:S9, A:Y10, A:I11, A:L15, A:N17, A:S18, A:R19, A:F20, A:G22, A:K23, A:N24, A:L25, A:G26, A:N27, A:Q28, A:Q29, A:S30, A:C31, A:Q32, A:D33, A:I34, A:N35, A:A36, A:S37, A:L38, A:F47, A:T48, A:S49, A:N61, A:V64, A:Q65, A:K66, A:G67, A:S76, A:E77, A:A78, A:A79, A:C80, A:T81, A:D82, A:G83, A:L84, A:P85, A:G86, A:V87, A:Q88, A:F89, A:A90, A:E91, A:R93, A:A94, A:S97, A:Y100, A:D101, A:Q104, A:V111, A:D112, A:L113, A:M114, A:N115, A:K116, A:C117, A:K118, A:I119, A:P120, A:L121, A:N122, A:N123, A:H142, A:V143, A:K144, A:K145, A:L146, A:I147, A:N148, A:K149, A:T150, A:M151, A:P152, A:P162, A:S163, A:F164, A:G165, A:S166, A:N167, A:K168, A:C169, A:E170, A:E171, A:R172, A:C174, A:S176, A:A178, A:K179	102	0.671
	Epitope 4	A:Q209, A:P210, A:A211, A:C212, A:S213, A:W214, A:Y215, A:N216	8	0.628
Sol g 2.2	Epitope 1	A:N2, A:E3, A:K6	3	0.88
	Epitope 2	A:A13, A:K14, A:A16, A:R17, A:T18, A:L19, A:P20, A:K21, A:C22, A:V23, A:N24, A:Q25, A:P26, A:D27, A:D28, A:R32	16	0.749
	Epitope 3	A:N48, A:P49, A:A50, A:P51, A:A52, A:V53, A:E56, A:R85, A:T87, A:Q88, A:R89, A:P90, A:R91, A:S92, A:N93, A:Q95, A:K96	17	0.723
	Epitope 4	A:I66, A:T67, A:D68, A:P69, A:A70, A:N71, A:E73, A:N74, A:K77	9	0.718
	Epitope 5	A:E4, A:V7, A:K10	3	0.597
	Epitope 6	A:T113, A:V114, A:L115, A:A116, A:R117, A:K119	6	0.531
Sol g 3.1	Epitope 1	A:T1, A:N2, A:Y3, A:C4, A:N5, A:L6, A:Q7, A:S8, A:C9, A:K10, A:R11, A:N12, A:N13, A:A14, A:I15, A:H16, A:C19, A:Q20, A:Y21, A:T22, A:S23, A:P24, A:T25, A:P26, A:G27, A:P28, A:L29, A:C30, A:L31, A:E32, A:C33, A:N94, A:Q95, A:C96, A:A102, A:C103, A:N105, A:S119, A:S120, A:S121, A:G122, A:E123, A:N124, A:K125, A:S126, A:F181, A:K182, A:E183, A:P184, A:D185, A:N186, A:W187, A:T188	53	0.739
	Epitope 2	A:Q55, A:K56, A:A58, A:S59, A:G60, A:K61, A:E62, A:M63, A:R64, A:G65, A:T66, A:N67, A:G68, A:Q69, A:Q70, A:P71, A:P72, A:A73, A:V74, A:N75, A:M76, A:P77, A:N78, A:L79, A:K140, A:D141, A:F142, A:D143, A:N144, A:R145, A:W146, A:S148, A:S149, A:F150, A:P151, A:S152, A:D153, A:P154, A:N155, A:I156, A:K159, A:A169, A:V202, A:L203, A:G204, A:A205, A:K206, A:Y208, A:E209, A:I210, A:K211, A:K212	52	0.659
	Epitope 3	A:F38, A:T39, A:D40, A:A41, A:D44, A:N48	6	0.631
	Epitope 4	A:R34, A:N35, A:V36	3	0.59
Sol g 4.1	Epitope 1	A:K22, A:G23, A:E24, A:N25, A:D26, A:P27, A:I28, A:N29, A:R33	9	0.773
	Epitope 2	A:E14, A:K15, A:I17, A:K18, A:T19, A:V20, A:P21	7	0.73
	Epitope 3	A:A1, A:D2, A:I3, A:K4, A:N7, A:I8, A:R11, A:E62, A:I65, A:I66, A:N67, A:P68, A:A69, A:N70, A:I71, A:K72, A:Q73	17	0.73
	Epitope 4	A:F47, A:T48, A:P49, A:K50, A:G51, A:K83, A:K84, A:V85, A:Y86, A:D87, A:R88, A:P89, A:G90, A:P91, A:I92, A:I93, A:E94, A:R95	18	0.694

^a^Protrusion Index (PI) values.

**Table 2:** The discontinuous B-cell epitope prediction of Pilosulins from ElliPro^30^.

Protein names	Epitope no	Residues	Number of residues	PI^a^ Score
Pilosulin 1	Epitope 1	A:Q54, A:P55, A:Q56	3	0.94
	Epitope 2	A:S49, A:Q50, A:E52, A:Q53	4	0.75
	Epitope 3	A:K23, A:L24, A:G25, A:P26, A:K27, A:V28	6	0.723
	Epitope 4	A:G1, A:L2, A:G3, A:S4, A:R8	5	0.6
Pilosulin 2	Epitope 1	A:D2, A:K4, A:K5	3	0.815
Pilosulin 3.2b	Epitope 1	A:G3, A:L4, A:V5	3	0.542
Pilosulin 4.1a	Epitope 1	A:F30, A:E31, A:K34	3	0.759
	Epitope 2	A:D2, A:K5, A:L6, A:N7, A:K9, A:K10	6	0.574
Pilosulin 5a	Epitope 1	A:D1, A:K3, A:G4, A:K7	4	0.797
	Epitope 2	A:E18, A:K19, A:G20, A:Y21, A:D22, A:K23	6	0.739

^a^Protrusion Index (PI) values.

Moreover, the predicted epitopes on Pilosulin proteins exhibited high allergic potential, particularly on Pilosulin 1 protein (PI score = 0.94, length = 3 amino acids). These included four epitopes that ranked as the top regions for B-cell epitopes, with Pilosulin 5a leading (PI = 0.797, 4 amino acids), followed by Pilosulin 4.1 (PI = 0.759), Pilosulin 2 (PI = 0.815, 3 amino acids), and Pilosulin 3.2b (PI = 0.542, 3 amino acids). Notably, this study revealed that the majority of the random coil and terminal regions in both Sol g proteins and Pilosulins were predicted as potential allergenic regions. However, the number of residues per epitope did not show a clear correlation with allergenicity or PI score prediction.

#### The in-silico epitope mapping and sequence alignments of Sol g protein and Pilosulins

The discontinuous B-cell epitopes were identified within the protein sequences of Sol g proteins, which were color-coded for clarity. Each Sol g protein sequence was aligned with Pilosulin protein sequences to highlight amino acid residues with potential cross-reactivity. The schematic diagram of Sol g 1.1 indicated a match with Pilosulins on Epitopes 3 and 4, encompassing 10 amino residues. Pilosulin 1 appeared to be a highly potential allergen for cross-reactivity with Sol g 1.1 (Fig. 3C). Sol g 2.2 was found to match with Pilosulins on epitopes 3 and 6, involving 11 amino residues (Fig. 3D). Additionally, Sol g 3.1 exhibited sequence alignment with Pilosulins (12 amino residues) on epitopes 1 and 2, while 4.1 proteins were specifically found on epitope 2. Notably, Pilosulin 4.1a displayed the highest number of amino residues matching with Sol g 3.1 (8 aa) and Sol g 4.1 sequence (9 aa) (Fig. 3E,F).

**Figure 4:**
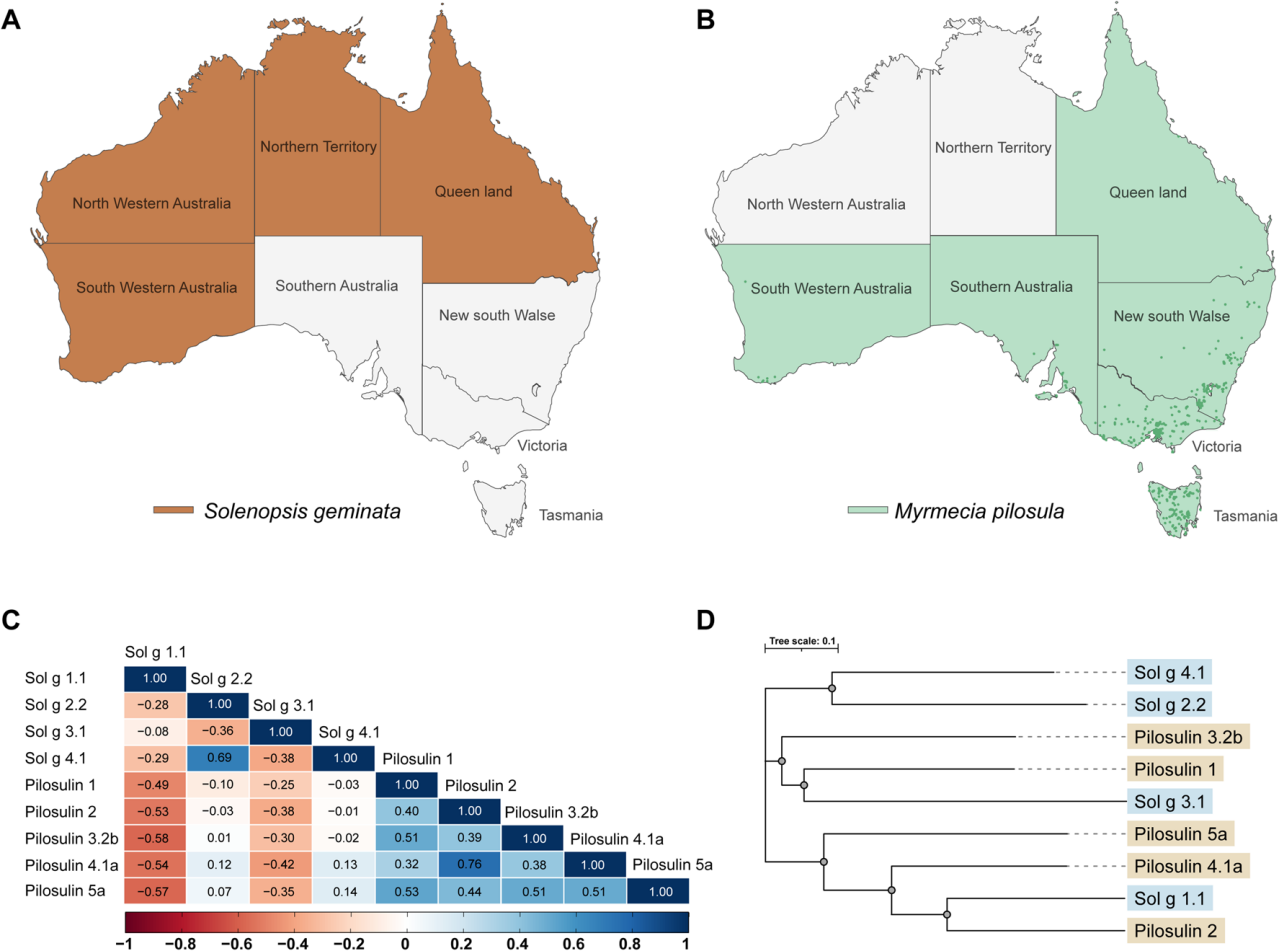
The distribution of (**A**) the invasive species Tropical fire ants (*Solenopsis geminata*) and (**B**) the native species *Myrmecia pilosula* in Australia (The Australia outline image was downloaded from https://mapsvg.com/maps/australia and modified using Adobe Illustrator version 25.0.1 (https://adobe.com/products/illustrator)). The relationship between the venoms of *S. geminata* and *M. pilosula* is demonstrated through (**C**) the BLOSUM62 correlation matrix and (**D**) the phylogenetic tree, which were constructed using the Clustal Omega tool for multiple sequence alignment.

The widespread distribution of *S. geminata* (Fig. 4A) in Australia may pose a high risk of cross-reactivity with native species (*M. pilosula*) (Fig. 4B). Therefore, sequence analysis was conducted for the primary interpretation of this study. Utilizing Clustal Omega for sequence alignment, the BLOSUM62 correlation matrix was applied to predict potential cross-reactions (Fig. 4C). Results indicated that Sol g 2.2 and Sol g 4.1 proteins posed a high risk of cross-reactive allergies with Pilosulins 4.1a and 5a, whereas Sol g 1.1 and 3.1 proteins showed a low risk. The phylogenetic tree illustrated the relatedness of the two allergic protein venoms (Fig. 4D). Three nodes represented distinct relationships among species. Sol g 1.1 is closely aligned with Pilosulin 2, forming a lineage with Pilosulin 4.1a and 5a. Additionally, Sol g 3.1 shared a relationship with Pilosulin 1 and 3.2b, exhibiting higher sequence similarity.

#### Specific IgE‐binding to recombinant Sol g proteins

**Figure 5:**
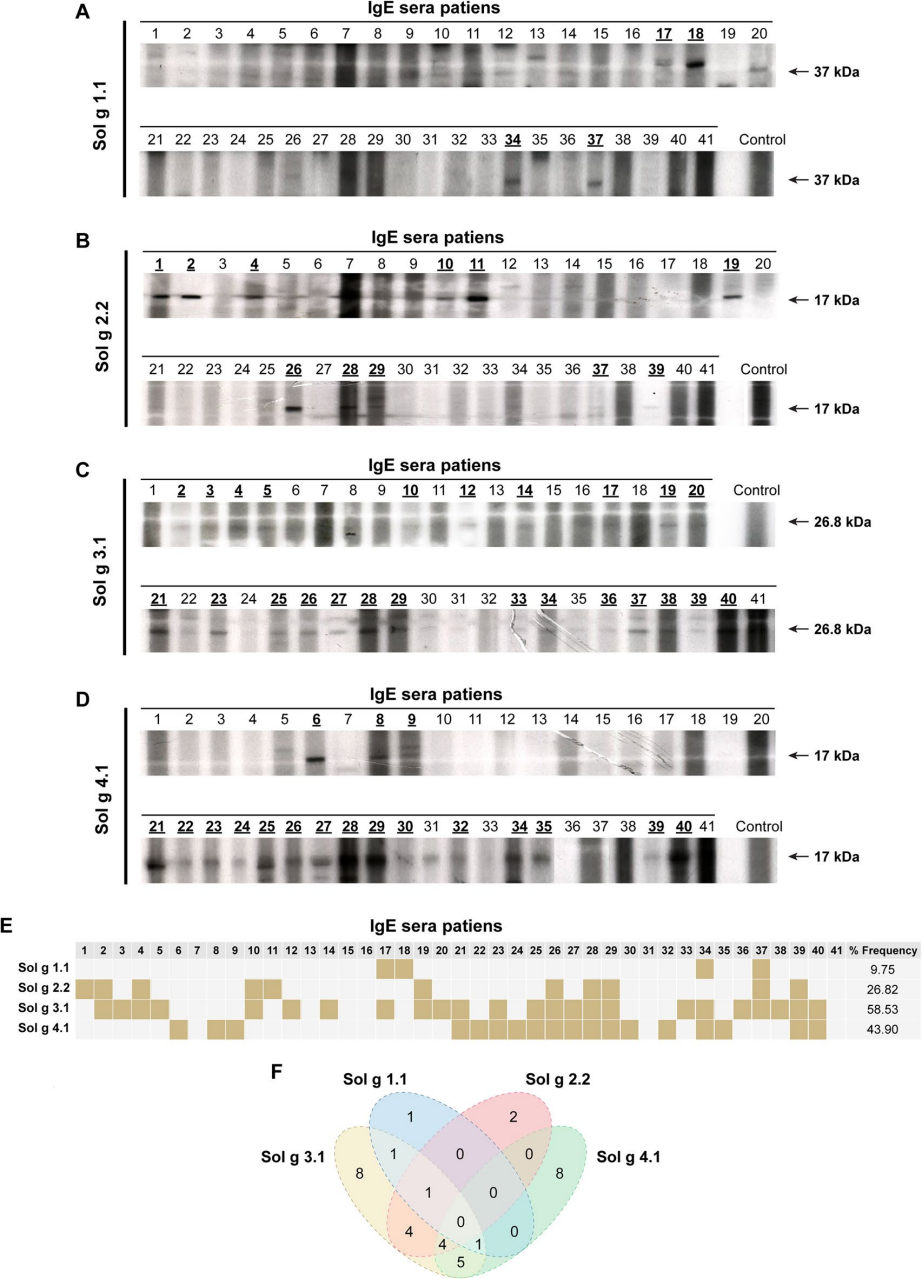
Immunoblotting was utilized to assess the serological cross-reactivity of sera obtained from patients allergic to *M. pilosula*. (**A**–**D**) The recombinant Sol g proteins were separated through SDS-PAGE and subsequently immunoblotted using IgE sera from individuals hypersensitive to *M. pilosula* venom. The positively reacting bands were highlighted numerically with bold and underlined styling. The western blot result image was composed by integrating cropped images and adding annotations. The unprocessed image is depicted in Supplementary File-Fig. 3. (**E**) The frequency of cross-reaction between recombinant Sol g proteins and *M. pilosula*-specific IgE was determined. Additionally, (**F**) The cross-reaction events were compared across individual patient sera.

All purified Sol g proteins were assessed for cross-reactivity through immunoblotting against sera from *M. pilosula* venom-allergic patients. Initially, SDS-PAGE was conducted to determine the sizes of the recombinant Sol g proteins. The r-Sol g 1.1 exhibited high-level expression at 37 kDa, whereas r-Sol g 3.1 was expressed at 26.8 kDa. Similar protein expressions were observed for r-Sol g 2.2 and r-Sol g 4.1, demonstrating sizes of 17 kDa each. Immunoblotting was then used to evaluate IgE cross-reactivity of the recombinant proteins against sera from 41 patients (Fig. 5A–D). Notably, both r-Sol g 3.1 and r-Sol g 4.1 proteins exhibited a high potential for cross-binding with sera from *M. pilosula* allergic patients, affecting 24 patients (58.53%) and 18 patients (43.90%), respectively (Fig. 5E).

Additionally, IgE cross-reactivity of r-Sol g 2.2 was identified in 11 patient sera (26.82%), while r-Sol g 1.1 protein displayed a low potential to bind with only 4 out of 41 (9.75%) IgE sera from venom-allergic patients. Furthermore, Venn diagrams illustrated the cross-reactive events for each patient's serum. The highest incidence of single cross-reaction events was observed with r-Sol g 3.1 and 4.1, whereas r-Sol g 2.2 and 1.1 showed a low potential for cross-reactivity (Fig. 5F). Double cross-reaction events were notably high for r-Sol g 3.1 when combined with 4.1, and for r-Sol g 3.1 when combined with 2.2. Moreover, r-Sol g 4.1 exhibited high double cross-reaction events with r-Sol g 3.1. Multiple cross-reaction events were prominent when r-Sol g 3.1 was combined with both r-Sol g 2.2 and 4.1, while the potential was low when combined with both r-Sol g 1.1 and 4.1.

## Discussion

The primary investigation into the cross-reaction of allergen-specific IgE antibodies with other allergen proteins commenced by analyzing the similarity of protein sequences. The sol g proteins exhibit a high sequence diversity, particularly in relation to other ant species associated with venom allergy, notably Sol g 1.1 and Sol g 3.1. Tropical fire ants (*S. geminata*) pose a significant threat as an invasive species that has expanded beyond their native region (South America)^31^. In our current study, particular attention is directed towards the extensive distribution of *S. geminata* in Australia, exerting numerous impacts on native species, ecosystems, and human health. The invasive ant is predominantly found in the North of Australia, while the native species (jack jumper ant (JJA); *M. pilosula*) is highly distributed in the South of Australia^32^. Reports on JJA-allergy cases indicate that 1.9% of adults in rural Victoria and 2.7% of the overall Tasmanian population are allergic to JJA venom^33,34^ . However, the cross-reactivity with tropical fire ant venom in patients allergic to Australian ants has not been investigated. The prevalence of cross-reaction between Hymenoptera allergens and their allergenic capacity has been reported in various studies^35,36^. This information is crucial for clinical diagnosis and therapeutic decisions^29^, with an emphasis on the potential cross-reaction of allergens reflecting patterns of clinical reactivity^25,26^. Limited studies have explored the cross-reaction of *S. geminata* allergens with other *Solenopsis* species, as well as cross-reactivity with non-related species. Both TFAs and JJA allergic proteins have obvious differences in the number of amino acids in their sequence and conformation of 3D structure. According to B-cell epitope prediction, we have emphasized conformational B-cell epitopes which are the main type of B-cell epitopes covering approximately 90% of the native proteins^37^. We discovered that B-cell epitopes on Sol g proteins and Pilosulins were predominantly located in random coil regions and specific positions on the alpha helix structure. Given the slightly complex 3D structure of Sol g proteins, which comprises numerous random coil regions, there is a high probability of the protein forming antigenic epitopes. Based on structure assessment, antigen 5 (Ag5) allergens, also known as catabolite activator protein (CAP) domain in vertebrates, from the venom of Hymenoptera is the most frequent trigger of severe IgE-mediated anaphylaxis in humans^38,39^. The Ag5 domain on the Sol g 3.1 structure (47–196 aa) was found on epitope 1 and epitope 2 of predicted conformation B-cell epitopes, while the Ag5 protein is absent in *M. pilosula* venom^40^. Furthermore, Phospholipase A1 (PLA1) is the predominant venom allergen of Vespoidea species relevant to allergies^41^. The PLA1 domain is also located on Sol g 1.1 and ranges from 33 to 215 aa, which was predicted to have 3 different epitope regions (epitope 2, 3, and 4). However, Sol g 2.2 and Sol g 4.1 shared a similar 3D protein structure, but there were differences in IgE binding capacity.

Sequence alignment methods are commonly employed for predicting cross-reactivity among allergenic proteins. Nevertheless, forecasting cross-reactivity between distantly related allergens remains a formidable challenge^42^. Allergen cross-reactivity typically occurs when IgE antibodies attach to either identical (with more than 70% sequence similarity) or closely resembling surface regions of another allergen that is related^26,28^. Less sequence identity or non-homologous allergens have not been comprehensively evaluated. The specificity of an antibody depends on the unique arrangement of amino acids at the epitope site, leading to potential cross-reactivity with analogous allergens from diverse sources^43,44^. Exploring cross-reactions among unrelated species is challenging, but our information reveals the relationship between Sol g proteins and Pilosulins through correlation matrices and phylogenetic trees. Sol g 3.1 protein is a focal point in our study due to its apparent high potential to cross-react with Pilosulins, especially Pilolusin 1 and 3.1. Additionally, Sol g 2.2 and 4.1, which share high sequence identity, also exhibit a strong relation to Pilosulin 4.1a and 5a. Cross-reaction analysis using the relation between amino acids within epitope sites could elucidate the possibility of sensitization with different sources of allergenic proteins.

This study represents the first report on the cross-reaction between *S. geminata* venom proteins and *M. pilosula* venom-specific IgE. The phenomenon of cross-reactivity among allergenic proteins is crucial for comprehending how the immune system identifies various antigen proteins. In addition, jack-jumper ant venom proteins may independently stimulate the production of IgE in individuals, each exhibiting distinct antigen epitope recognition on these allergens. Various factors, such as genetic predisposition, environmental exposure, and prior sensitization, can influence an individual's IgE response to allergens^45^. While people may differ in their sensitivity to specific allergens, two or more individuals can share similar levels of IgE recognition for the same allergen. Immunoblotting indicates high cross-reaction of Sol g 3.1 and 4.1 proteins, which were recognized by the IgE of 58.53% and 43.90%, respectively of *M. pilosula* allergic patients. Additionally, cross-reaction events involving Sol g 3.1 with Sol g 4.1 or 2.2 were observed, while Sol g 1.1 showed a low likelihood of double or multiple cross-reaction events. Cross-reaction may occur randomly through Sol g proteins and is not specific to a particular type of them. Consequently, even though the patients were exposed to an identical allergen (*M. pilosula* venom), their IgE exhibited recognition of different epitopes. This discrepancy in epitope recognition is responsible for the varying affinities and cross-reactivity of individual patients' IgE to four types of recombinant Sol g proteins. Our data reveal cross-reaction in 35 cases of the total patient sera analyzed, posing a high risk (85.36%) for future reactions in patients exposed to *S. geminata* venom. Therefore, Sol g 3.1 and Sol g 4.1 in *S. geminata* venom could be major allergenic proteins inducing allergic reactions in *M. pilosula*-sensitized patients.

In summary, our findings suggest the presence of IgE antibodies with specific affinity to jack jumper ant venom, exhibiting cross-reactivity with other unrelated major allergens found in fire ant venom. This phenomenon poses a risk of promoting allergen aggregation, IgE-mediated mast cell activation, and is a leading cause of anaphylaxis and other allergic symptoms. This comprehensive understanding is crucial for individuals allergic to *M. pilosula* venom to prevent exposure to *S. geminata* venom. Exploration of cross-reactivities among non-related allergens in Hymenoptera venoms is necessary for future allergy prevention strategies, especially for individuals with existing severe allergies.

## Conclusion

This study's significant revelation about cross-reactivity among unrelated allergenic venoms will have substantial implications for diagnosis and immunotherapy. It demonstrates the antibody cross-reaction potential of non-homologous allergen venoms between jack jumper ant venom (*M. pilosula*) and fire ant venom (*S. geminata*). This event may be responsible for the heightened allergenic potency observed in individuals allergic to *M. pilosula* venom. Therefore, this study serves as a foundational knowledge base for understanding the propensity for cross-reactivity among unrelated allergenic venoms in Hymenoptera. It provides a reliable basis for designing future therapeutic strategies.

## Methods

### Fire ant venom collection and gland extraction

All animal research protocols were reviewed and approved by the Animal Ethics Committee of Khon Kaen University, Thailand (No. AEKKU 54/2556). This study adhered to the ARRIVE guidelines and the guidelines for the Care and Use of Animals for Scientific Purposes as outlined by the National Research Council of Thailand. The venom extracted from the stinger tips of *S. geminata* workers in suburban Khon Kaen City, Khon Kaen Province, Thailand, was collected using capillary tubes under a magnifying glass. Additionally, the abdominal part of *S. geminata* was homogenized with PBS buffer at a ratio of 1:200 w/v. The soluble protein was obtained by centrifuging at 10,000 rpm for 10 min and then stored at − 80 °C for future use. Protein content was quantified using the Bradford method^46^, with bovine serum albumin serving as the standard.

### Isolation of RNA and cDNA synthesis

Total RNA was isolated from the entire bodies of *S. geminata* workers using TRIzol reagent (Invitrogen, Life Technologies, USA), following the manufacturer's protocol. Absorbance measurements at 260 nm and 280 nm were conducted to evaluate the RNA sample quality. Subsequently, the synthesis of complementary DNA (cDNA) was carried out using the RevertAid First Strand cDNA Synthesis Kit (Thermo Scientific, USA) following the manufacturer's instructions.

### Identification of Sol g proteins by sequencings 3’- and 5’-RACE (Rapid Amplification of cDNA Ends)

The RACE procedures were performed using the RACE System according to the manufacturer's instructions of Invitrogen, Life Technologies. Both 3’ and 5’-RACE reactions utilized the PCR master mix reagent kit (Fermentas, Singapore) with Taq DNA polymerase. The PCR consisted of 30 cycles, each comprising 30 s at 94 °C, 1 min at 58 °C, and 1 min at 72 °C. A final polymerization step was carried out for 7 min. For Nested PCR, the amplicon from the initial PCR reaction served as the template. The resulting PCR products, which possessed a single A overhang at the 3’ end, were cloned into a vector with compatible T-overhangs (pGEM-T Easy vector, Promega, USA) for Edman degradation sequencing.

### In silico protein structure prediction and sequence analysis

The Sol g protein sequences were fully translated into protein sequences using the Translate tool (https://web.expasy.org/translate/) (Supplementary File-Tables S2, S3). Meanwhile, the amino acid sequences of Pilosulin proteins (*M. pilosula*) were obtained from prior studies (Supplementary File-Table S4)^19–22^. Protein structure prediction utilized AlphaFold2, accessible on https://colab.research.google.com/github/sokrypton/ColabFold/blob/main/AlphaFold2.ipynb, accessed on 20 September 2023. The predicted local distance difference test (pLDDT) served as the confidence measure for model predictions. Additionally, the error in the position of each amino acid was calculated based on the predicted aligned error (PAE), with parameters set to 20 recycles and a root mean square deviation (RMSD) tolerance of 0.5 Å. Single chain predictions were ranked by pLDDT, and complexes were ranked by predicted TM-score. The five models generated for each run, along with their predicted pTM and pLDDT scores, were collected. Furthermore, the features of predicted modes were obtained from SWISS-MODEL (https://swissmodel.expasy.org/), which are homologous to experimentally determined structures of related families in the database. Refinement and annotation were performed using Chimera UCSF software (version 1.6.1)^47^.

### Conformational B-cell epitope prediction on antigen protein structures

The 3D structural models of allergic proteins, obtained in AlphaFold's PDB format, were utilized to predict conformational (discontinuous) B-cell epitopes. This prediction was based on specific threshold values for the protrusion index *S* (minimum score) and the distance *R* (maximum distance) between the centers of mass for each residue. The parameters were configured as *S* = 0.5 and *R* = 6 Å for the computation of discontinuous epitopes using ElliPro (http://tools.iedb.org/ellipro/). Additionally, the surface of B-cell epitopes was delineated on the predicted 3D structures of Sol g proteins and Pilosulins using ChimeraX (version 1.6.1). The schematic representation of B-cell epitopes was visualized alongside a histogram, considering scores exceeding 0.5 as the threshold value.

### The Blosum62 correlation matrix and phylogenetic tree analysis

The multiple sequence alignment (MSA) data were acquired from BLASTP (2.14.1) to compare the similarity between the protein query and the protein databases in Unipro Ugene software (version 48.1)^48^. The BLOSUM62 alignment score matrix was computed and visualized using RStudio software (version 2022.07.1) to investigate the correlation among protein sequences. Additionally, a phylogenetic tree specifically, the Neighbour-Joining tree without distance corrections was established using Clustal Omega (https://www.ebi.ac.uk/jdispatcher/msa/clustalo) and visualized with iTOL (Interactive Tree of Life) (https://itol.embl.de).

### Molecular cloning, expression, purification, and IgE cross-reactivity of recombinant Sol g Proteins PCR amplification

The PCR amplification of mature gene fragments for Sol g 1.1, 2.2, 3.1, and 4.1 was carried out using specific primers (Table 3.) that incorporated restriction enzymes based on the nucleotide sequencing of these genes. The PCR reaction mixture was prepared according to the manufacturer's instructions for GoTaq Green Master Mix (Promega, USA). Additionally, a negative control, in which the master mix was replaced with double-distilled H_2_O, was included. The PCR cycling profile for all reactions was as follows: initial denaturation at 94 °C for 5 min, template denaturation at 94 °C for 30 s, annealing at 58 °C for 30 s, and extension at 72 °C for 1 min. This cycle was repeated for 30 cycles, with a final extension at 72 °C for 7 min. The resulting PCR products were confirmed by electrophoresis on a 1% Tris–Acetate EDTA (TAE) agarose gel.

**Table 3:** Specific primers were designed with a restriction enzyme for the expected PCR amplification of the Sol g genes.

Gene	Sense	Primer sequences (5′–3ʹ)	Product size (bp)
Sol g 1.1	Forward (Sol1F_Bam)	TATGGATCCCAGTCCTGTGTCTACGGTAATTCTAGC	948
	Reverse (Sol1R_Pst)	ATTCTGCAGTTATTGCTGCCTGCACTTAAATAGATTTTGTGC	
Sol g 2.2	Forward (Sol2F_Eco)	ACGTGGAATTCAACATAATGAAGAACTAAAAGTTATACAT	366
	Reverse (Sol2R_Pst)	ATTCTGCAGTCATTTTTTACGGGCTAGCACTGTAGTCTCC	
Sol g 3.1	Forward (Sol3F_Eco)	CTAGAATTCAAACAAATTATTGCAACCTTCAATCATGTAAGAG	693
	Reverse (Sol3R_Pst)	TATCTGCAGCTATTTCTTTATTTCGTATATTTTTGCACCCAGC	
Sol g 4.1	Forward (FSol4_Nc)	CCATGGCTGCTGATATTAAGGA	369
	Reverse (RSol4_Xho)	CTCGAGTCATTTTTTTTTGCCATACACTG	

### Gel purification

The desired PCR amplicons were purified using a PureLink Quick Gel Extraction Kit in accordance with the manufacturer’s instructions (Invitrogen, USA). In brief, a solubilization buffer (1.2 mL) was applied to the gel slice containing the amplicon and incubated at 50 °C for 10 min. The dissolved gel piece was then transferred into the column and centrifuged at 12,000 × g for 1 min, followed by a washing step. To collect the purified PCR product, elution buffer was added and centrifuged. Finally, the purified product was subjected to a second run on a 1% TAE agarose gel to confirm successful recovery.

### Cloning and expression

The PCR fragments of interest were incorporated into TOPO vectors through the utilization of the TOPO TA Cloning Kit (Invitrogen, USA). Subsequently, the resulting recombinant plasmid was introduced into DH5α *E. coli* competent cells, and the transformants were screened with 100 μg/mL ampicillin on LB agar plates. Afterward, the identified positive colonies were selected and cultivated in LB medium containing 100 μg/mL ampicillin at 37 °C overnight, and the plasmid was then isolated and purified using the Presto Mini Plasmid Kit (Geneaid, Taiwan).

The construction of the recombinant plasmid involved the deployment of the pProEX-HTB vector, which encompasses ampicillin resistance selectivity and an N-terminal Hisx6 tag. Initially, the pProEX-HTB vector underwent double digestion with restriction enzymes, specifically BamHI and PstI for Sol g 1.1, EcoRI and PstI for Sol g 2.2 and 3.1, and NcoI and XhoI for Sol g 4.1. Following this, the purified PCR products were ligated in frame into the digested pProEX-HTB expression vector and subsequently transformed into DH5α *E. coli* competent cells.

Analysis of the recombinant proteins involved SDS-PAGE, and the expression of proteins was verified through immuno-blotting using goat anti-mouse IgG-linked horse radish peroxidase (HRP). Finally, purification of the His-tagged recombinant protein was executed using the HisPur Ni–NTA resin purification system (Thermo Fisher Scientific, USA), followed by SDS-PAGE and Western blot analysis as previously described.

### IgE cross-reactivity of recombinant Sol g proteins against IgE sera from allergic patients

The experiments involving humans were conducted in accordance with the guidelines of the Declaration of Helsinki and received ethical approval from both the Royal Hobart Hospital and Flinders Medical Centre ethics committees^49^. All participants with a confirmed history of allergic reactions to jack jumper ant (*Myrmecia* spp.) provided written informed consent before blood sample collection. Their epidemiological and serum data are detailed in Table 4. and Supplementary File-Table S5.

**Table 4:** Demographic characteristics of study patients with a history of allergic reactions to jack jumper ant (*M. pilosula*) venom.

Characteristic	*n* = 41
1. Gender	
Male	14
Female	27
2. Age-year	
< 18	3 (7.31%)
18–60	11 (26.82%)
≥ 60	17 (41.46%)
N/A	10
Mean ± SD^a^	54.48 ± 18.63
3. Total IgE (kU/l)	
Mean	1.43 ± 5.11
IQR	0.63

*IQR* interquartile range.

^a^Mean ± standard deviation.

Firstly, r-Sol g proteins were separated on a 13% SDS-PAGE gel, then transferred to an activated PVDF membrane (BioRad), and blocked with a solution of 5% skim milk powder in PBS containing 0.05% Tween-20 as described previously. Subsequently, the membrane underwent overnight incubation with patient sera diluted at a ratio of 1:10 using a slot blot apparatus (Idea Scientific, USA)^50^. IgE binding was detected using a rabbit anti-human IgE polyclonal antibody labeled with horseradish peroxidase, diluted at a ratio of 1:2500 (DAKO Corporation, USA). Following washing with PBST buffer (PBS with Tween 20), the blots were incubated for 5 min with 1 mL of Pierce Western blotting enhanced chemiluminescence (ECL) substrate (Thermo Fisher Scientific, USA). Protein bands were visualized by exposure to ECL Hyperfilm (GE Healthcare Biosciences, UK) and developed using standard X-ray film development techniques.

### Supplementary Information

Supplementary Information.

## Data Availability

The structure of Phospholipase A1 from *Solenopsis invicta* is available on the UniProt repository with the PDB ID Q68KK0. The detailed code for Alphafold can be found at https://colab.research.google.com/github/sokrypton/ColabFold/blob/main/AlphaFold2.ipynb. The datasets generated during this study are accessible in the Zenodo repository at https://doi.org/10.5281/zenodo.10608631.
