# Supplementary material for: Structure-based epitope prediction and assessment of cross-reactivity of Myrmecia pilosula venom-specific IgE and recombinant Sol g proteins (Solenopsis geminata)
Source: Sci Rep. 2024 May 15;14:11145. doi: 10.1038/s41598-024-61843-4 (PMC11096326; doi:10.1038/s41598-024-61843-4)
Supplement: Supplementary file 1 — Supplementary Information. [file 41598_2024_61843_MOESM1_ESM.docx]

**
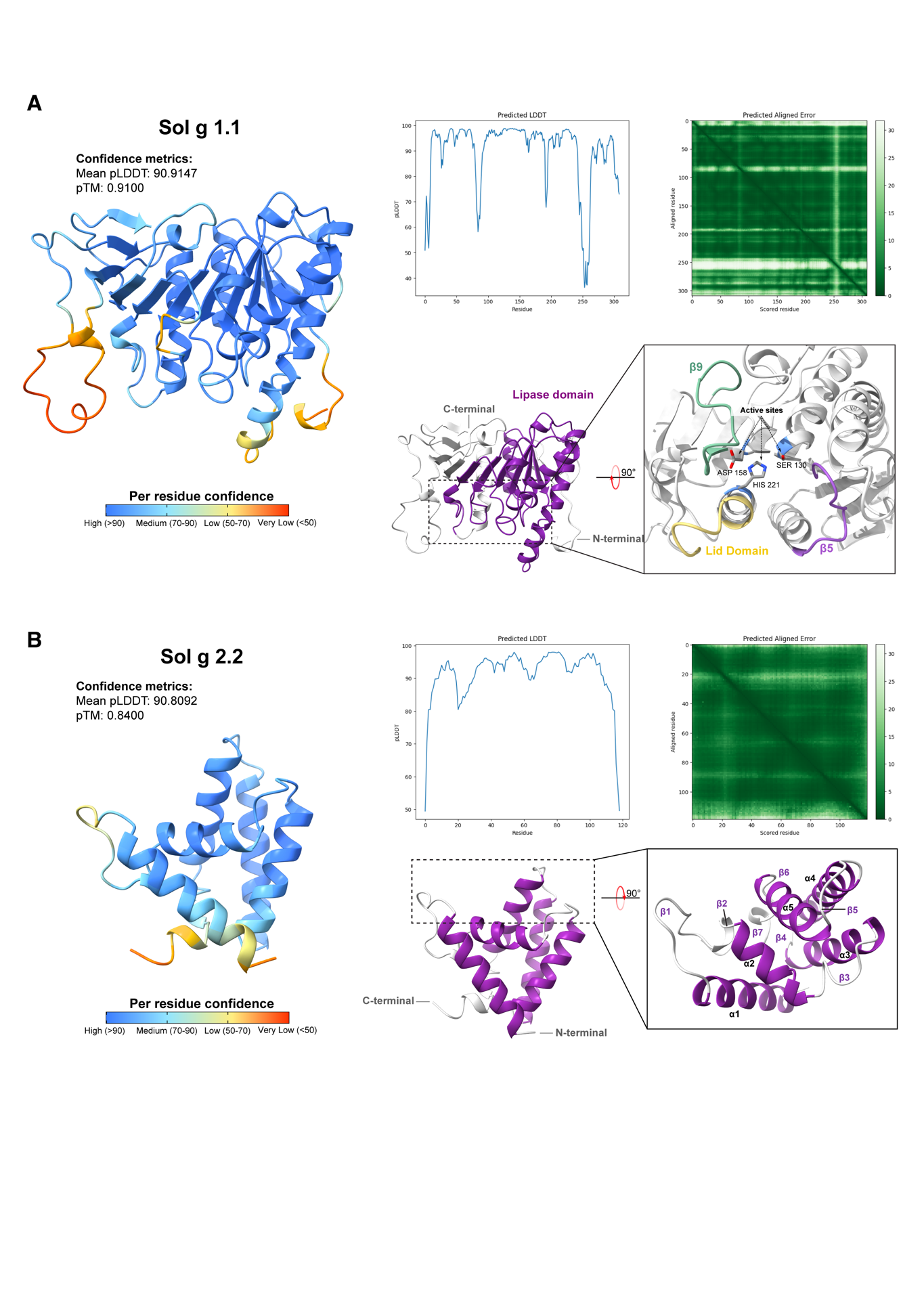
**

**Supplementary File-Figure 1.** The AlphaFold structure predictions of (**A**) Sol g 1.1 and (**B**) Sol g 2.2 proteins include confidence quality scores represented as pLDDT and pTM. The confidence residues of the models were colored based on the pLDDT score, with blue indicating high confidence and red indicating lower confidence. The amino acid positions were plotted against the predicted LDDT to estimate confidence on a scale from 0 to 100, while the predicted aligned error (PAE) provides a distance error for every pair of residues, ranging from 0 Å (dark green) to 33 Å (white)


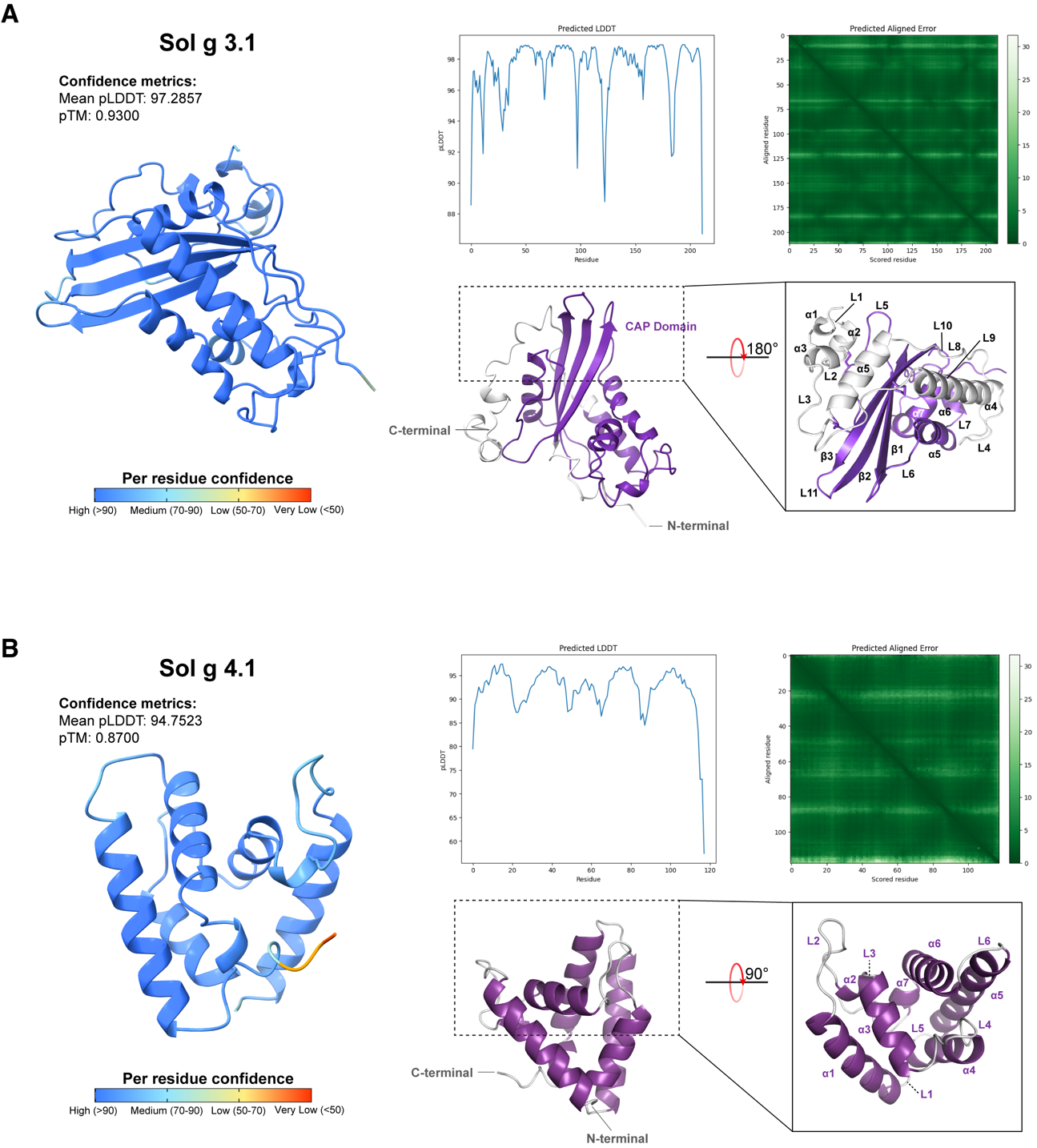


**Supplementary File-Figure 2.** The AlphaFold structure predictions of (**A**) Sol g 3.1 and (**B**) Sol g 4.1 proteins include confidence quality scores represented as pLDDT and pTM. The confidence residues of the models were colored based on the pLDDT score, with blue indicating high confidence and red indicating lower confidence. The amino acid positions were plotted against the predicted LDDT to estimate confidence on a scale from 0 to 100, while the predicted aligned error (PAE) provides a distance error for every pair of residues, ranging from 0 Å (dark green) to 33 Å (white)

**Supplementary File-Table 1.** The degenerative primer design and PCR products for Sol g proteins.

| Sol G protein genes | Forward primers | Reverse primers | Product size (bp) |
| --- | --- | --- | --- |
| Sol g 1.1 | Fmg1  5’-RAGYCTGATSCGGGG-3’ | Rmg1  5’-TDATTKCTGCCTGS-3’ | 963 |
| Sol g 2.2 | Fpg2  5΄-GACCCAGMTCCAGCTGC-3΄ | Rmg2 5΄-TCATTTTTYACGRGCTAGC-3΄ | 417 |
| Sol g 3.1 | Fmg3  5’-ASAARTTATHGCAACCTTY-3’ | Rmg3  5’-CTAWTTCKTTAYTBCG-3’ | 639 |
| Sol g 4.1 | F2  5 ́-AAWGTATRAAWACAVHAYC-3 ́ | R3  5 ́-CKTYTBYCAWTKATTRGTSCC-3 ́ | 414 |

**Abbreviations**; R = purine (A or G), Y = pyrimidine (C or T), B = not A (C, G, or T), K = keto (G or T), S = strong (C or G), M = amino (A or C), and W = weak (A or T).

**Supplementary File-Table 2.** The DNA sequences of Sol g proteins were obtained from Edman degradation sequencing.

| **Sol G protein genes** | **DNA sequences (5’-3’)** |
| --- | --- |
| Sol g 1.1  (963 amino acids) | GAGCCTGATCCGGGGGTTGTAGAGTATTTGAAACAGTCCTGTGTCTACGGTAATTCTAGCTACATTAACGTATACTTGTACAACAGTCGTTTCCAGGGCAAAAATCTCGGGAACCAACAGAGCTGCCAGGATATTAATGCATCACTACCGGTTGTCTTCATAACTCATGGCTTCACCAGTTCTGCACAAGTGTCTACATTTAAAGATCTGGCAAATGCATTTGTACAGAAAGGTCATACAGCATTTATAGTAGATTGGTCTGAGGCAGCTTGCACTGATGGACTGCCCGGTGTTCAATTTGCCGAGTATAGGGCGGCGGCATCGAACACTTATGATATCGGTCAACTTATGGCAAAATATACCGTTGATTTGATGAACAAGTGTAAAATTCCATTGAATAATATCCAATACGTAGGTCACAGTCTTGGCTCACACGTGTGCGGCTTTGCTGCAAAACATGTTAAAAAATTGATAAACAAAACCATGCCGTATATCTTAGCACTCGATCCTGCTGATCCTTCCTTCGGTAGCAATAAGTGCGAGGAAAGAATCTGCAAGAGTGATGCCAAACGTATAGTAGTTTTTAAGACATCGATTCTCGGAATAGGGAAGAATATTATAGGCCACCTCCTCATAGTGTTCGATGGTGGGAAATCGCAACCGGCTTGTTCATGGTATAACGTTCCTTGCTCACACAGCGAAAGTATCGTATATGCAACCGGGATGGTGAGTGGTAGATGTCAACACCTTGCTGTTCCTTGGACAGCTCAGCAGAGAATCAACCCAATTCAATGGAAATTCTGGAGAGTTTTCACATCAAATATACCCGCTTATCCTACCTCTGACACAAAGAATTGTGTAGTTTTGAACACCAATGTATTTAAGAATGACAATACTTTCGAAGGAGAATACCACGCTTTTCCTGACTGTGCACAAAATCTATTTAAGTGCAGGCAGCAATAA |
| Sol g 2.2  (417 amino acids) | ATGAAGTCCTTTGTGTTTGCTACATGTCTGCTAGTTTTTGCGCAGATCATTTACGCACATAATGAAGAACTAAAAGTTATACATAAGGATATAGCAAAATGTGCAAGAACATTACCAAAATGCGTAAATCAACCAGATGATCCGTTAGCTAGAGTCGATGTATGGCATTGTGCTATGGCCAAGCGTGGCGTATATGACAACCCAGCTCCAGCTGTTATAAAAGAAAAAAATTTTAAAGTATGCTCCAAGATTATCACTGATCCCGCTAATGTCGAAAATTGCAAGAAAGTTATTTCTAGATGTGTAGATAGAGAGACTCAACGCCCAAGATCCAACAGACAGAAAGCAATAAATATAACAGGATGTATTTTAAGAGCTGGTGTGGCGGAGACTACAGTGCTAGCCCGTAAAAAATGA |
| Sol g 3.1  (639 amino acids) | ACAAATTATTGCAACCTTCAATCATGTAAGAGAAACAATGCAATTCATACGATGTGCCAATATACCTCACCAACACCGGGGCCATTGTGCTTGGAATGTAGAAATGTAGGTTTTACTGACGCTGAGAAAGATGCTATTGTAAACAAACACAATGAACTGAGGCAGAAAGTCGCATCGGGTAAGGAAATGAGAGGAACGAACGGCCAGCAACCTCCGGCGGTTAATATGCCGAATTTGACCTGGGATCCAGAATTAGAGATGATTGCTCAAAGATGGGCTAATCAGTGCATGGATGAACATGATGCTTGTAGAAACGTAGAAAGATTCGCAGTAGGTCAGAATATAGCTTCAACATCTAGCTCGGGTGAAAATAAGTCAACTGTAAATGACATGATTTTATTATGGTACGATGAAGTAAAAGACTTCGATAATCGTTGGATTTCCTCATTTCCATCTGACCCTAACATTCTGATGAAAATAGGACATTATACTCAAATTGTTTGGGCTAAAACGGTGAAAATCGGGTGTGGACGAATAATGTTCAAGGAACCAGATAACTGGACTAAACATTATTTAGTTTGCAACTATGGTCCAGCTGGAAATGTGCTGGGTGCAAAAATATACGAAATAAAGAAATAG |
| Sol g 4.1  (414 amino acids) | ATGAAAACCTTCGTGCTTGCTACATGTCTGCTAGTGTTTACGCAGATAATTTACGCAGCTGATATTAAGGAAATAAATATCATGAATAGAATTTTAGAAAAATGTATAAAAACAGTACCAAAAGGCGAAAATGATCCAATAAATCCTTTGAGAAGAGTCAATGTGTGGTATTGTACACTCACTAAGCGTGGCATATTTACTCCAAAAGGTGTAAATACGAAACAATATATTAGCTATTGCGAAAAGACGATCATTAATCCTGCTAATATAAAACAGTGCAAGAAATTAGTTTCTAAATGCATAAAGAAAGTGTATGACCGCCCGGGACCAATCATTGAGAGAAGCAAAAATCTATTGTCATGTGTTATAAAAAAGGGTGTGCTCGAATTGACAGTGTATGGCAAAAAAAAATGA |

**Supplementary File-Table 3.** The putative amino sequences of Sol g proteins were translated from the Translate tool (<https://web.expasy.org/translate/>).

| **Sol g proteins** | **Protein sequences** |
| --- | --- |
| Sol g 1.1  (320 amino acids) | EPDPGVVEYLKQSCVYGNSSYINVYLYNSRFQGKNLGNQQSCQDINASLPVVFITHGFTSSAQVSTFKDLANAFVQKGHTAFIVDWSEAACTDGLPGVQFAEYRAAASNTYDIGQLMAKYTVDLMNKCKIPLNNIQYVGHSLGSHVCGFAAKHVKKLINKTMPYILALDPADPSFGSNKCEERICKSDAKRIVVFKTSILGIGKNIIGHLLIVFDGGKSQPACSWYNVPCSHSESIVYATGMVSGRCQHLAVPWTAQQRINPIQWKFWRVFTSNIPAYPTSDTKNCVVLNTNVFKNDNTFEGEYHAFPDCAQNLFKCRQQ |
| Sol g 2.2  (138 amino acids) | MKSFVFATCLLVFAQIIYAHNEELKVIHKDIAKCARTLPKCVNQPDDPLARVDVWHCAMAKRGVYDNPAPAVIKEKNFKVCSKIITDPANVENCKKVISRCVDRETQRPRSNRQKAINITGCILRAGVAETTVLARKK |
| Sol g 3.1  (212 amino acids) | TNYCNLQSCKRNNAIHTMCQYTSPTPGPLCLECRNVGFTDAEKDAIVNKHNELRQKVASGKEMRGTNGQQPPAVNMPNLTWDPELEMIAQRWANQCMDEHDACRNVERFAVGQNIASTSSSGENKSTVNDMILLWYDEVKDFDNRWISSFPSDPNILMKIGHYTQIVWAKTVKIGCGRIMFKEPDNWTKHYLVCNYGPAGNVLGAKIYEIKK |
| Sol g 4.1  (137 amino acids) | MKTFVLATCLLVFTQIIYAADIKEINIMNRILEKCIKTVPKGENDPINPLRRVNVWYCTLTKRGIFTPKGVNTKQYISYCEKTIINPANIKQCKKLVSKCIKKVYDRPGPIIERSKNLLSCVIKKGVLELTVYGKKK |

* The underline indicates the putative signal sequences.

**Supplementary File-Table 4.** The amino sequences of Pilosulin proteins from jack jumper venom (*Myrmecia pilosula*).

| **Pilosulin proteins** | **Protein sequences** |
| --- | --- |
| Pilosulin 1^a^  (56 amino acids) | GLGSVFGRLARILGRVIPKVAKKLGPKVAKVLPKVMKEAIPMAVEMAKSQEEQQPQ |
| Pilosulin 2^b^  (27 amino acids) | IDWKKVDWKKVSKKTCKVMLKACKFLG |
| Pilosulin 3.2b^c^  (24 amino acids) | IIGLVSKGTCVLVKTVCKKVLKQG |
| Pilosulin 4.1a^d^  (36 amino acids) | FDITKLNIKKLTKATCKVISKGASMCKVLFEKKKQE |
| Pilosulin 5a^c^  (37 amino acids) | DVKGMKKAIKEILDCVIEKGYDKLAAKLKKVIQQLWE |

^a^; Donovan *et al*. (1993), ^b^; Donovan and Baldo (1997) & Donovan *et al*. (1993), ^c^; Inagaki *et al*. (2008), ^d^; Wiese *et al*. (2007)

**Supplementary File-Table 5.** Demographic characteristics of study patients with a confirmed history of allergic reactions to jack jumper ant (*M. pilosula*)

| **No.** | **ID** | **Sex** | **AGE** | **Date of collection** | **Total IgE (kU/l)** |
| --- | --- | --- | --- | --- | --- |
| 1 | AVAS 31 | F | N/A | N/A | 32.48164165 |
| 2 | AVAS 107 | M | N/A | N/A | 0.016030434 |
| 3 | AVAS 133 | F | 69 | 11/05/2006 | 1.510272564 |
| 4 | AVAS 135 | F | 76 | 09/10/2006 | 0 |
| 5 | AVAS 145 | F | N/A | N/A | 0.025990273 |
| 6 | AVAS 148 | M | N/A | N/A | 0.017122479 |
| 7 | AVAS 160 | F | 62 | 05/04/2006 | 0.64817099 |
| 8 | AVAS 161 | F | N/A | N/A | 0.156998872 |
| 9 | AVAS 181 | M | N/A | N/A | 0.091450522 |
| 10 | AVAS 217 | F | 35 | 29/03/2006 | 5.489756652 |
| 11 | AVAS 229 | M | 69 | 20/03/2006 | 0.645975906 |
| 12 | AVAS 252 | F | 66 | 24/07/2006 | 0.228152262 |
| 13 | AVAS 261 | M | 67 | 03/07/2006 | 0.012571878 |
| 14 | AVAS 263 | F | 42 | 05/06/2006 | 0.900107048 |
| 15 | AVAS 265 | M | 55 | 11/05/2006 | 0.03270469 |
| 16 | AVAS 318 | F | 31 | 13/12/2006 | 0 |
| 17 | AVAS 330 | F | 66 | 23/01/2007 | 2.313045024 |
| 18 | AVAS 335 | F | 31 | 15/12/2006 | 2.226468559 |
| 19 | AVAS 336 | F | N/A | N/A | 0.205619105 |
| 20 | AVAS 342 | M | 8 | 20/02/2007 | 1.883215528 |
| 21 | AVAS 343 | M | 70 | 06/02/2007 | 0 |
| 22 | AVAS 357 | F | N/A | N/A | 0.090183818 |
| 23 | AVAS 367 | F | 15 | 14/04/2007 | 0.017301677 |
| 24 | AVAS 376 | F | 76 | 16/03/2007 | 0 |
| 25 | AVAS 377 | F | 75 | 19/03/2007 | 0 |
| 26 | AVAS 419 | F | 61 | 30/04/2007 | 0 |
| 27 | AVAS 422 | F | 62 | 23/04/2007 | 0.135026968 |
| 28 | AVAS 423 | M | N/A | N/A | 0.518949715 |
| 29 | AVAS 432 | F | 66 | 07/05/2007 | 0.034904407 |
| 30 | AVAS 443 | M | N/A | N/A | 2.082836039 |
| 31 | AVAS 452 | F | 55 | 04/04/2007 | 0.032083358 |
| 32 | AVAS 512 | M | 15 | 09/11/2007 | 0.169507179 |
| 33 | AVAS 517 | F | 56 | 26/11/2008 | 0 |
| 34 | AVAS 520 | F | 65 | 27/02/2008 | 0.098901185 |
| 35 | AVAS 545 | F | 57 | 26/01/2008 | 0.059781496 |
| 36 | AVAS 617 | F | 37 | 14/03/2008 | 1.944689325 |
| 37 | AVAS 619 | M | 50 | 10/04/2008 | 0.089691923 |
| 38 | AVAS 621 | M | 68 | 21/05/2008 | 0.032958281 |
| 39 | AVAS 624 | M | 60 | 01/05/2008 | 0 |
| 40 | AVAS 625 | F | 62 | 21/05/2008 | 0.272145522 |
| 41 | AVAS 626 | F | 62 | 09/05/2008 | 4.563747585 |


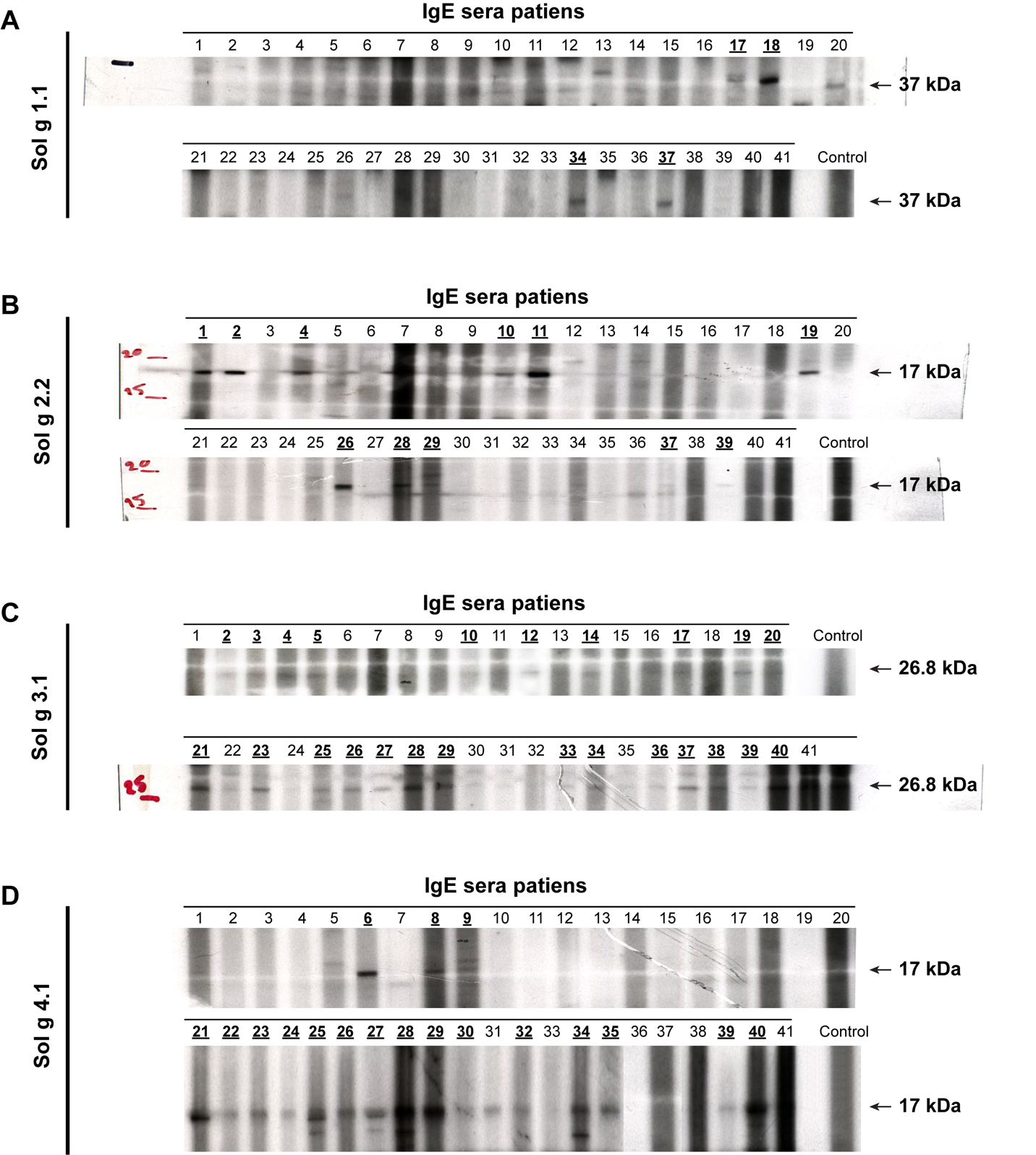


**Supplementary File-Figure 3.** Immunoblotting was used to examine the serological cross-reactivity of sera collected from patients allergic to *M. pilosula*. Recombinant Sol g proteins (A-D) were separated via SDS-PAGE and then immunoblotted with IgE sera from individuals sensitive to *M. pilosula* venom. The protein bands were detected by exposing them to ECL Hyperfilm from GE Healthcare Biosciences (UK) and developing them using standard X-ray film development methods. The bands that showed a positive reaction were identified with numerical highlights in bold and underlined formatting.
